# Patient Characteristics and Reasons for Switching to Bictegravir/Emtricitabine/Tenofovir Alafenamide: A Retrospective Cohort From a Large Urban HIV Clinic

**DOI:** 10.7759/cureus.95684

**Published:** 2025-10-29

**Authors:** Hao Gao, Bhavya Manchukonda, Pedro Simoes, Eleanor Hamlyn, Tristan J Barber, Alan Hunter, Jennifer Hart, Margaret Johnson, Sabine Kinloch-de Loes

**Affiliations:** 1 Acute Medicine Department, King's College Hospital NHS Foundation Trust, London, GBR; 2 The Ian Charleson Day Centre, Royal Free London NHS Foundation Trust, London, GBR; 3 Institute of Global Health, Royal Free London NHS Foundation Trust, London, GBR; 4 Department of Virology, Royal Free London NHS Foundation Trust, London, GBR

**Keywords:** antiretroviral therapy (art), b/f/taf, bictegravir, human immunodeficiency virus (hiv), integrase strand transfer inhibitor (insti), nhs, simplification, toxicity, treatment switch

## Abstract

Introduction

Second-generation integrase strand transfer inhibitor (INSTI)-containing regimens have been preferred in almost all international guidelines for the treatment of human immunodeficiency virus-1 (HIV-1) since the early 2010s. This study aimed to determine patient characteristics and primary reasons for switching to a bictegravir/emtricitabine/tenofovir alafenamide (B/F/TAF) regimen in a large, inner-city cohort of people living with HIV-1 in London, United Kingdom (UK), and to assess the impact of the 2022 UK National Procurement Policy on prescribing patterns.

Methods

A retrospective review of electronic patient records was conducted for all individuals on antiretroviral therapy (ART) who switched to B/F/TAF at the Royal Free Hospital, London, United Kingdom. Sociodemographic data, clinical markers including HIV-1 viral load, CD4^+^ T cell count, and previous ART regimen, and the documented reason for the switch were collected. Data were censored on 20 November 2022, allowing for a comparison of switch frequency before and after the implementation of the UK National Procurement for HIV Treatment and Prevention on 1 February 2022. Data collection was completed in the timeframe of 20 November 2022 till 30 June 2023.

Results

The cohort included 386 individuals who switched to B/F/TAF, of whom 74.1% were male, 25.9% were female, 56% were White, and 27% were African/Caribbean, with a median age of 58.5 years. Median duration of living with diagnosed HIV-1 and ART exposure prior to the switch were both 20 years. The most common reason for switching was regimen simplification (n=169, 43.8%), followed by toxicity (n=115, 29.8%); nearly 10% (n=38, 9.8%) switched due to virological failure. Number of switches increased from 74 in the observed period from April-November 2021 to 168 between April-November 2022 following the implementation of the National Procurement Policy.

Conclusions

The primary reason for switching to B/F/TAF was the desire for regimen simplification, a trend that appears to have been reinforced by the National Procurement Policy. However, toxicity, adherence issues, and virological failure were also key clinical motivations for switching. These findings highlight the role of B/F/TAF in ART optimisation for a diverse and treatment-experienced population of people living with HIV.

## Introduction

Human immunodeficiency virus (HIV) infection, if left untreated, can lead to the development of acquired immunodeficiency syndrome (AIDS) and death in most individuals [1]. Combination antiretroviral therapy (ART) has dramatically transformed its prognosis with the control of viraemia, immune reconstitution, and decrease of onward transmission [1].

Historically, several classes of ART agents have been combined, such as two nucleoside reverse transcriptase inhibitors (NRTIs) with either protease inhibitors (PIs) or non-nucleoside reverse transcriptase inhibitors (NNRTIs). However, these regimens often came with challenges, such as requiring multiple daily doses, stringent food restrictions, and adverse effects that have remained a central issue [2]. Switching ART regimens is a key clinical strategy for optimising long-term treatment, with goals that include simplifying a regimen to reduce pill burden and improve adherence, or mitigating adverse effects by improving the metabolic profile and reducing drug toxicities.

First-generation integrase strand transfer inhibitors (INSTIs), such as raltegravir, have demonstrated major advances, with fewer drug-drug interactions (DDIs) and high tolerability and effectiveness, but have a low genetic resistance [3]. Second-generation INSTIs (dolutegravir, bictegravir, and cabotegravir), with the latter used as an injectable treatment in association with rilpivirine, combine high potency, efficacy, and tolerability with a high barrier to resistance, proving further superiority to previous regimens [4].

This shift has also been reflected in changes to guidelines, as the British HIV Association (BHIVA) initially recommended raltegravir in 2012, then elvitegravir-cobicistat in 2013, and ultimately dolutegravir in 2015 [5,6]. Second-generation INSTIs have since become the preferred first-line treatment choice for adolescents and adults with HIV-1 as per national and international guidelines [7-9].

Bictegravir is an INSTI with a high genetic barrier to the development of HIV-1 resistance. It is administered as a once-daily tablet with a fixed-dose combination of bictegravir/emtricitabine/tenofovir alafenamide (B/F/TAF) as the trade name of Biktarvy®. It was approved in the United States in February 2018 and in the European Union in June 2018. The U.S. Food and Drug Administration (FDA) approved it based on the clinical efficacy demonstrated in four clinical trials involving adults with HIV-1 infection, with two trials conducted in naïve individuals and two in virologically controlled individuals [10-13].

The primary objectives of the study were to describe the sociodemographic and clinical characteristics of individuals in a large, real-world UK cohort switching to a B/F/TAF regimen and to identify and quantify the primary reasons for these treatment switches. The secondary objective was to assess the impact of the 2022 UK National Procurement Policy on the frequency of switches to B/F/TAF.

This article was previously presented as an abstract and poster at the BHIVA Spring 2023 Conference on April 24, 2023.

## Materials and methods

This study was conducted and reported in accordance with the Strengthening the Reporting of Observational Studies in Epidemiology (STROBE) guidelines for cohort studies (Appendix).

This is a retrospective cohort study using electronic patient records of a cohort of people living with HIV in a large inner-city hospital, the Royal Free Hospital, London, United Kingdom, to review all those on ART who had switched from existing regimens to B/F/TAF and data from the Royal Free HIV Cohort Study (RFHCS), which has been running since the early 1990s [14]. The RFHCS is a single-centre, observational study of people attending for HIV care at the Royal Free Hospital. It includes sociodemographic characteristics (such as date of birth, gender, and ethnicity), mode of transmission, date of diagnosis of HIV-1 infection, initial HIV-1 viral load and CD4+ T cell count, longitudinal values, all ART regimens and reasons for switching regimens, and resistance data. Data were censored on 20 November 2022. Data collection was completed in the timeframe of 20 November 2022 till 30 June 2023.

The study population included all individuals in the RFHCS who switched their ART regimen to B/F/TAF within the study period. The only exclusion criterion was the absence of a documented reason for the switch in the electronic patient record, which would constitute incomplete data for the primary outcome. For our analysis, we extracted from the RFHCS baseline characteristics such as gender, ethnicity, age of people living with HIV who had switched to B/F/TAF, mode of transmission, median CD4+ T cell count stratified by gender, follow-up from date of switch to censor date, duration of diagnosed HIV-1 infection at the time of switch, ART duration prior to switch, ART regimen prior to switch, duration of switch, category of toxicity leading to switching, and numbers of switches and reasons for switches. We also compared the number of switches to B/F/TAF during two periods (April 2021 to November 2021 and April 2022 to November 2022), which were before and after the National Procurement for HIV Treatment and Prevention.

As the aim of this study was descriptive, data are presented as counts and percentages or as medians and interquartile ranges (IQRs). Therefore, formal statistical comparisons with confidence intervals or other measures of uncertainty were not performed. Powered analysis was also not applicable in this study as it was a retrospective observational study.

To mitigate potential biases inherent in a retrospective study, sever steps were taken. Selection bias was minimised by including all eligible individuals who switched to B/F/TAF at our centre during the study period, ensuring a comprehensive sample. To address potential information bias from electronic records, data on the reasons for switching were extracted systematically and categorised according to a pre-defined classification system applied consistently across all records. While confounding by indication - where the clinical reason for a switch may also be related to outcomes - cannot be fully eliminated in an observational design, we collected comprehensive baseline demographic and clinical data to thoroughly characterise the cohort.

## Results

The study cohort consists of 386 people living with HIV-1 who had switched their ART regimen to B/F/TAF. Of these, 286 were males and 100 females, accounting for 74.1% and 25.9% of individuals, respectively. The total number of individuals under follow-up at the Royal Free Hospital in 2022 was 3,170 people living with HIV.

**Figure 1:**
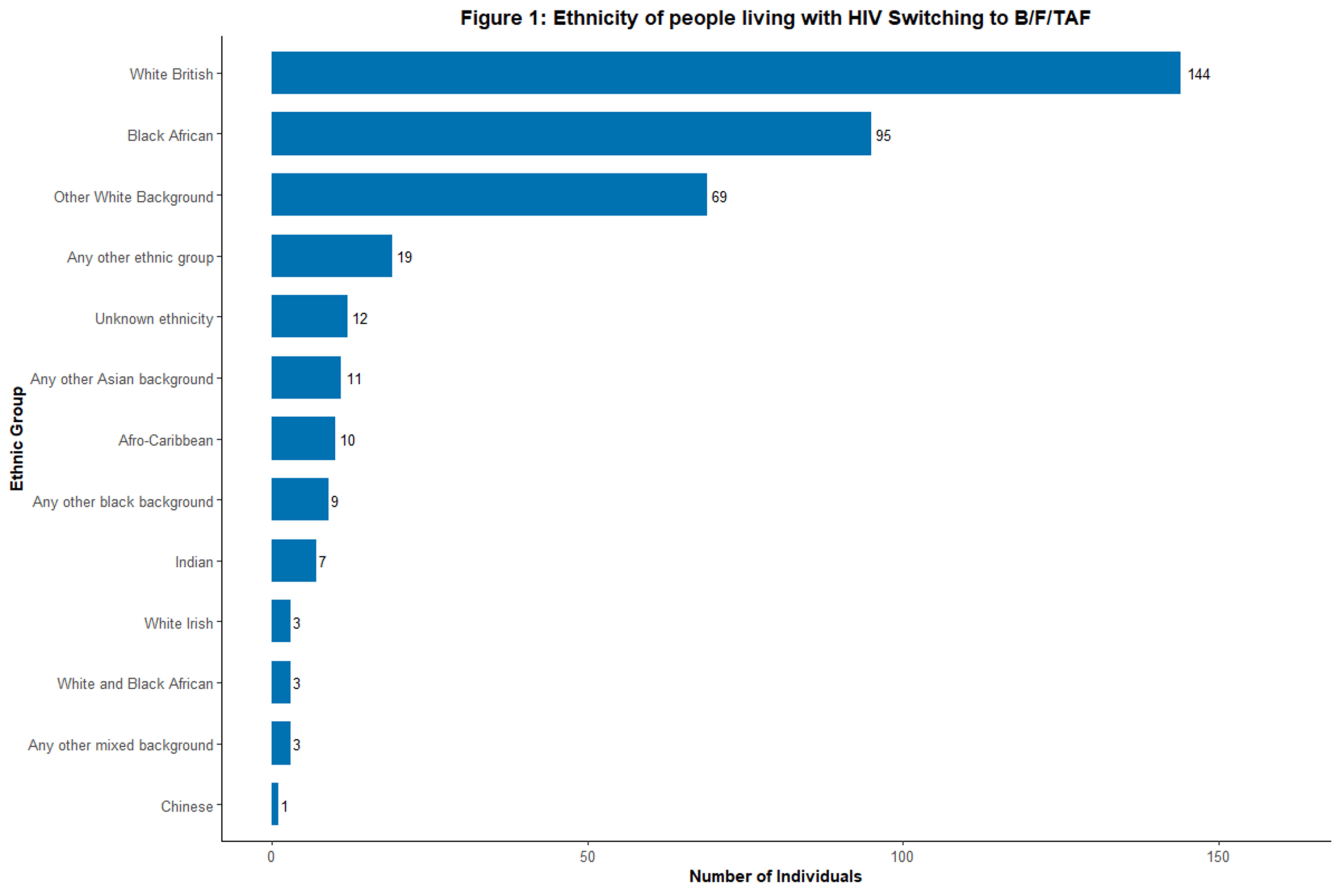
Ethnicity of people living with HIV switching to B/F/TAF (n=386) The horizontal bar chart displays the number of individuals within each self-reported ethnic group who switched to a B/F/TAF regimen. B/F/TAF, bictegravir/emtricitabine/tenofovir alafenamide

Figure 1 illustrates the ethnicity of people living with HIV who switched to B/F/TAF. Leading ethnicities included 144 white British (37.3%) and 95 individuals of black African descent (24.6%). The next largest group was of other white backgrounds, with 69 (17.9%) individuals. Other ethnic groups included the following: 19 (4.9%) of any other ethnic group, 11 (2.8%) of any other Asian background, 10 (2.6%) of Afro-Caribbean background, 9 (2.3%) of any other black background, 7 of Indian background (1.8%), 3 (0.8%) of White Irish background, 3 (0.8%) of White and Black African background, 1 (0.3%) of Chinese background, 3 (0.8%) of any other mixed background, and 12 of unknown ethnicity (3.1%).

Median age for the entire cohort was 58.5 years (IQR 53-63) with a median age of 59 years for men (IQR 53-64) and 57 years for women (IQR 52.5-62). The most common transmission risk was sex between men in 194 individuals (50.3%), followed by heterosexual sexual transmission in 150 (38.9%) individuals. Other modes of transmission included shared syringes or needles in 10 (2.6%) individuals, blood transfusions in 7 (1.8%) individuals, oral sex in 2 (0.5%) individuals, vertical transmission in 1 (0.3%) individuals, and unknown mode of transmission in 22 (5.7% individuals) (note: percentages do not total 100 due to rounding).

Median CD4+ T cell count at switch was 541 cells/mm3 (IQR 380-701.5 cells/mm3). Median CD4+ T cell count at switch was 562 cells/mm3 for women (IQR 427-736 cells mm3) and 530 cells/mm3 for men (IQR 377-676 cells mm3). Median follow-up from the date of B/F/TAF switch to censor was nine months (IQR 5-19 months), and median duration of diagnosed HIV-1 infection and ART exposure prior to switch was 20 years (IQR 14-27 years).

**Figure 2:**
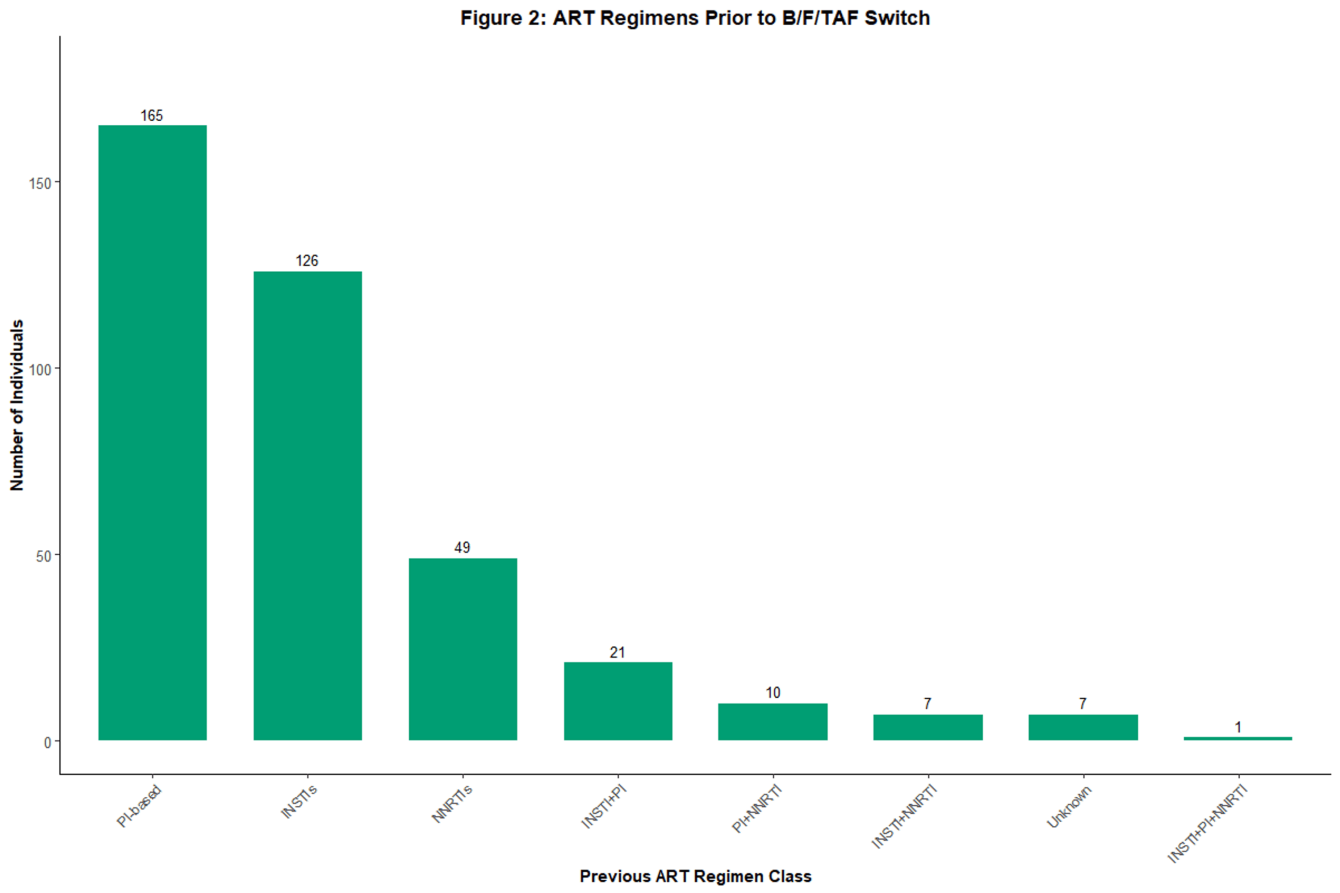
ART regimens prior to B/F/TAF switch (n=386) The vertical bar chart shows the number of individuals switching from each class of ART. ART, antiretroviral therapy; B/F/TAF, bictegravir/emtricitabine/tenofovir alafenamide; INSTI, integrase strand transfer inhibitor; NNRTI, non-nucleoside reverse transcriptase inhibitor; PI, protease inhibitor

Figure 2 shows ART regimens prior to the B/F/TAF switch, the most common of which was a PI-based regimen in 165 (42.7%) individuals. INSTIs were the second most common regimen, with 126 (32.6%) individuals. Other regimens included NNRTIs in 49 (12.7%) individuals, INSTI+PI in 21 (5.4%) individuals, PI+NNRTI in 10 (2.6%) individuals, INSTI+NNRTI in 7 (1.8%) individuals, INSTI+PI+NNRTI regimen in 1 (0.3%) individuals, and unknown prior regimens in 7 (1.8%) individuals (note: percentages do not total 100 due to rounding).

**Figure 3:**
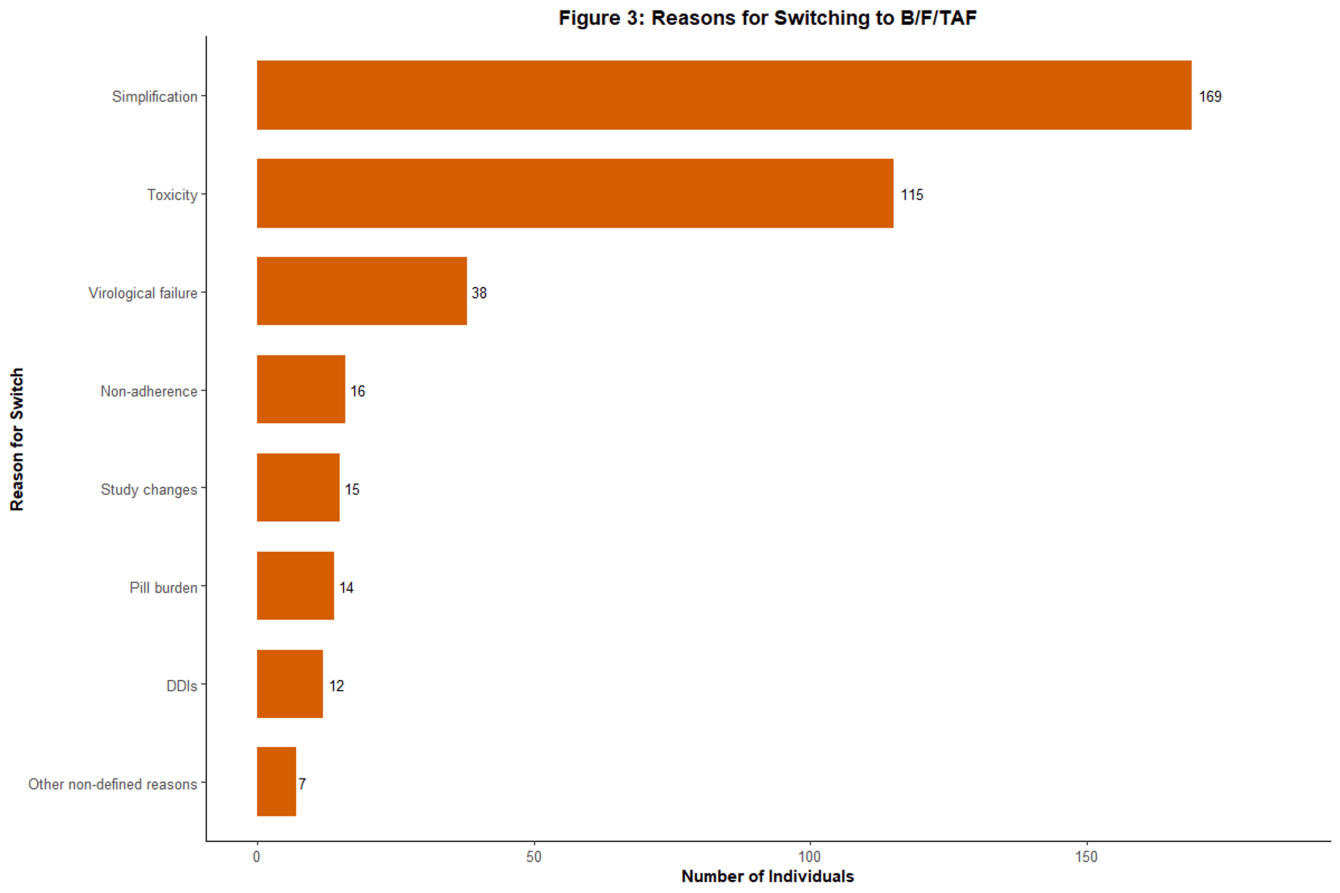
Reasons for switching to B/F/TAF (n=386) The horizontal bar chart displays the primary documented reasons for switching from a prior ART regimen. ART, antiretroviral therapy; B/F/TAF, bictegravir/emtricitabine/tenofovir alafenamide; DDI, drug-drug interaction

Figure 3 shows the reasons for the B/F/TAF switch. The most common one was simplification of the current regimen, with 169 (43.8%) individuals. The next most common reason was regimen toxicity, with 115 (29.8%) individuals. Virological failure (defined as a viral load ≥ 50 copies/mL) was a reason for the switch in 38 (9.8%) individuals, and non-adherence to the previous regimen was a reason in 16 (4.1%) individuals. Pill burden was the reason for switch in 14 (3.6%) individuals. The remaining cohort had other reasons for switching: 12 (3.1%) individuals had DDIs, 15 (3.9%) individuals had study changes, and 7 (1.8%) individuals had other non-defined reasons (note: percentages do not total 100 due to rounding).

We have further divided toxicity into specific categories (Table 1). These include 40 (34.8%) switches due to renal toxicity, 19 (16.5%) due to hyperlipidaemia, 17 (14.8%) due to bone toxicity, 11 (9.6%) due to central nervous system toxicity, 10 (8.7%) due to gastrointestinal toxicity, 5 (4.3%) due to mixed toxicity, and 13 (11.3%) due to unknown toxicity type.

**Table 1:** Toxicity categories leading to switch (n=115) The table details the breakdown of clinician-reported toxicities from previous ART regimens that prompted a switch to B/F/TAF. ART, antiretroviral therapy; B/F/TAF, bictegravir/emtricitabine/tenofovir alafenamide

Type of Toxicity	Number of Individuals (n=115)	Percentage (%)
Renal toxicity	40	34.8
Hyperlipidaemia	19	16.5
Bone toxicity	17	14.8
Central nervous system	11	9.6
Gastrointestinal	10	8.7
Mixed toxicity	5	4.3
Unknown	13	11.3

Of the 386 total switches, 74 occurred between April 2021 and November 2021 and 168 occurred between April 2022 and November 2022. The remaining 144 switches took place either before April 2021 or during the interval from December 2021 to March 2022.

## Discussion

We describe in this study the population characteristics and reasons for a switch to a bictegravir-based ART regimen from a previous regimen in our clinic cohort of people living with HIV-1 in the UK. The main results showed that the primary reason for switching to B/F/TAF was simplification/rationalisation (43.8%), followed by toxicity (29.8%). For 38 (9.9%) out of 386 the reason for switch was detectable viraemia on the previous regimen. This may have been due to a variety of reasons including adherence, multiple tablets, tolerability, or underlying/undetected previous resistance. The sociodemographic profile of the switching cohort was consistent with that of the larger clinic population. There can be various explanations for the fact that simplification/rationalisation was the most cited primary reason for switching seen in our cohort. One such example might be the larger number of pills, such as with a PI-based regimen, in comparison to the single tablet regimen with B/F/TAF. Furthermore, simplification is a key strategy for mitigating the risk of DDIs, a critical consideration in an ageing cohort with increasing polypharmacy due to comorbidities.

The effectiveness of B/F/TAF in individuals of different demographic backgrounds has also been studied, such as in black Americans [15], women [16], and elderly people [17]. These studies showed that B/F/TAF is non-inferior to other ART regimens and has a good safety profile with potent antiviral effect, allowing the prescribing of B/F/TAF to reach a diverse population. This, in turn, would have justified the safety of the switches.

One key finding in this study was the drastic increase in the number of switches in the period pre- compared to the post-implementation of the NHS England National Procurement for HIV Treatment and Prevention beginning on 1 February 2022. The change in policy allowed patients in England to have access to the same HIV medications across regions [18]. The accompanying guidance also explicitly encouraged clinicians to review patient medications and discuss switching to different treatments as appropriate, as part of a shared decision-making process [18]. Our results in patients undergoing regimen switches before and after the policy change are likely to reflect this change. This suggests how a national healthcare policy may directly influence and shape front-line prescribing patterns.

This study found that the largest group of patients (42.7%) switched from a PI-based regimen. This is significant when considering the potential positive metabolic implications of switching to an INSTI-based regimen; however, INSTIs as a class have been suggested to be associated with weight gain, particularly when compared to older NNRTIs or regimens containing tenofovir disoproxil fumarate [19]. However, this is not applicable in switches from a PI-based regimen to an INSTI-based regimen, given that no significant weight changes were noted in such switches [19]. In addition, studies have shown that switching away from PI-based regimens can lead to a reduction in serum lipid levels [17, 20], a named toxicity recorded in our study.

Results from this study also revealed that 9.8% of the cohort (38 individuals) switched to B/F/TAF due to virological failure. Their regimens prior to switching to B/F/TAF included INSTI, INSTI-PI, INSTI-NRTI, NNRTI, and PI. The three main pre-switch regimens were PI, INSTI, and NNRTI, respectively. Data from a pooled study showed good long-term efficacy with B/F/TAF, including in patients with pre-existing NRTI resistance mutations such as M184V/I, one of the most common forms of resistance [21]. Another study with pooled analyses of clinical trials also showed that over 90% of viraemic events (defined as a viral load ≥ 50 copies/mL) are followed by rapid virological re-suppression while continuing B/F/TAF, with a median time to re-suppression of just 22 days [22].

The reasons for switching have also evolved over time with ART development and newer treatment options. An Italian switch study in 2012-2015 showed that, in the earlier years of the epidemic, reasons to switch ART were mainly due to virological failure, whereas in a later period, simplification was, as in our study, the main reason for a switch [23]. Another study has suggested that simplification and toxicity was one of the major reasons for switches [24]. As more treatment options become available, new challenges are also emerging with ageing cohorts, comorbidities, and polypharmacy, which may now dictate switches.

When considering switches due to toxicity in 29.8% of our cohort (115 individuals), renal toxicity was by far the most common reason. This suggests that B/F/TAF was perceived to have a better track record with renal impairment related to ART as compared to tenofovir-based regimens, although a creatinine clearance below 30 mL/minute remains a contraindication to TAF use [25].

There are limitations to our study that we must acknowledge, particularly due to its retrospective design and reliance on electronic patient records for data collection. This means that although we can show strong correlations, we cannot definitively prove causality. Another limitation lies in the type and availability of the data collection from day-to-day clinical care, which suggests that the underlying clinical decision-making for a regimen switch may have been more complex than those listed in the dataset. Being a single-centre study also means that the generalisability of our results may be limited. However, this focus also provides details in a real-world clinical practice setting with a large and diverse cohort. We hope that studies from other centres demonstrating similar results would increase the generalisability of our study results. Furthermore, with a relatively short median follow-up period of nine months, although sufficient to capture the immediate reasons for switching regimens and any early trends, the timeframe also results in a less comprehensive observation of long-term outcomes. The findings from this study provide a baseline for a future analysis, which will assess long-term outcomes of these switches following the full implementation of the new ongoing NHS England clinical commissioning policy regarding tenofovir alafenamide [26].

Building on these limitations, it is important to acknowledge the specific sources of bias that could influence our findings. Selection bias may be present, as our single-centre cohort, while large, may not be fully representative of all people living with HIV in the UK, potentially limiting the generalisability of our results. Information bias is also a consideration, as the documented reasons for switching can vary in detail between clinicians. Future prospective, multi-centre studies could mitigate these biases by employing standardised, protocol-driven data collection and incorporating advanced statistical methods to better control for confounding variables.

## Conclusions

From the data collected, we can see that the primary driver for switching to a fixed-dose regimen of B/F/TAF was drug regimen simplification. This trend coincided with the implementation of a National HIV regimen Procurement Policy and was likely reinforced by its guidance encouraging clinicians to review and optimise treatments.

## Appendices

**Table 2:** STROBE Checklist Strobe Checklist: Checklist of items recommended by the STROBE statement for cohort studies. The "Recommendation & Location" column indicates the page and section where each item is addressed within this paper. *Give information separately for exposed and unexposed groups. ART, antiretroviral therapy; B/F/TAF, bictegravir/emtricitabine/tenofovir alafenamide; INSTIs, integrase strand transfer inhibitor; RFHCS, Royal Free HIV Cohort Study; STROBE, Strengthening the Reporting of Observational Studies in Epidemiology

	Item No.	Recommendation and Location
Title and abstract	1	(a) Indicate the study’s design with a commonly used term in the title or the abstract. Location: Title (“…A Retrospective Cohort from a Large Urban HIV Clinic”); Page 1, Abstract, Method (“A retrospective review of electronic patient records was conducted…”)
		(b) Provide in the abstract an informative and balanced summary of what was done and what was found. Location (Page 1, Abstract): The abstract provides a structured summary of the study’s introduction, methods, key results (cohort size, demographics, primary reasons for switch, policy impact), and conclusions.
Introduction		
Background/rationale	2	Explain the scientific background and rationale for the investigation being reported. Location (Page 1-2, Introduction): This section details the evolution of ART, the development and advantages of second-generation INSTIs, and the approval of B/F/TAF, establishing the rationale for a real-world study on switching patterns.
Objectives	3	State specific objectives, including any prespecified hypotheses. Location (Page 2, Introduction, Paragraph 6): The objectives are clearly stated, covering the characterisation of the cohort, the reasons for switching, and the impact of the national procurement policy.
Methods		
Study design	4	Present key elements of study design early in the paper. Location (Page 2, Materials and Methods, Paragraph 1): “This is a retrospective cohort study using electronic patient records…”
Setting	5	Describe the setting, locations, and relevant dates, including periods of recruitment, exposure, follow-up, and data collection. Location (Page 2, Materials and Methods, Paragraphs 1 and 2): The setting is identified as the Royal Free Hospital, London, UK. The data censorship date (20 November 2022), data collection timeframe (20 November 2022 to 30 June 2023), and comparison periods are all specified.
Participants	6	(a) Give the eligibility criteria, and the sources and methods of selection of participants. Describe methods of follow-up. Location (Page 2, Materials and Methods, Paragraph 3): Eligibility is defined as all people in the RFHCS who switched to B/F/TAF. The source is the RFHCS electronic records. Follow-up is described by the fixed data censorship date.
		(b) For matched studies, give matching criteria and number of exposed and unexposed groups. Not applicable: This was not a matched study.
Variables	7	Clearly define all outcomes, exposures, predictors, potential confounders, and effect modifiers. Give diagnostic criteria, if applicable. Location (Page 2, Materials and Methods, Paragraph 4): The primary outcomes (reasons for switch) and key variables (demographics, clinical markers, prior ART) are clearly listed. Virological failure is defined in the Results section (Page 4, Paragraph 1).
Data sources/measurement	8*	For each variable of interest, give sources of data and details of methods of assessment (measurement). Describe comparability of assessment methods if there is more than one group. Location (Page 2, Materials and Methods, Paragraphs 1 and 2): The source for all variables is explicitly stated as “electronic patient records” from “The Royal Free HIV Cohort Study (RFHCS).”
Bias	9	Describe any efforts to address potential sources of bias. Location (Page 2, Materials and Methods, Final Paragraph): This section details the steps taken to mitigate selection bias (inclusion of all eligible patients) and information bias (systematic data extraction and categorisation).
Study size	10	Explain how the study size was arrived at. Location (Page 2, Materials and Methods, Paragraph 3): The sample size was determined by including all eligible patients within the defined timeframe.
Quantitative variables	11	Explain how quantitative variables were handled in the analyses. If applicable, describe which groupings were chosen and why. Location (Page 2, Materials and Methods, Paragraph 4): “As the aim of this study was descriptive, data are presented as counts and percentages or medians and interquartile ranges.”
Statistical methods	12	(a) Describe all statistical methods, including those used to control for confounding.
		(b) Describe any methods used to examine subgroups and interactions.
		(c) Explain how missing data were addressed.
		(d) If applicable, explain how loss to follow-up was addressed.
		(e) Describe any sensitivity analyses. Location (Page 2, Materials and Methods, Paragraph 4): (a) The study is descriptive; no formal statistical methods for confounding were used. (b) Subgroup data is presented descriptively (e.g., CD4 count by gender). (c) Missing data are handled by including categories such as “unknown ethnicity” and “unknown prior regimens.” (d) Not applicable in the traditional sense due to the retrospective design with a fixed censor date. (e) No sensitivity analyses were performed.
Results		
Participants	13*	(a) Report numbers of individuals at each stage of study, e.g., numbers potentially eligible, examined for eligibility, confirmed eligible, included in the study, completing follow-up, and analysed.
		(b) Give reasons for non-participation at each stage.
		(c) Consider use of a flow diagram. Location (Page 3, Results, Paragraph 1): (a) The final number of individuals included (n=386) and the total number in the hospital cohort (n=3170) are reported. (b) The Methods section states that cases with incomplete data were excluded. (c) A flow diagram was not used.
Descriptive data	14*	(a) Give characteristics of study participants (e.g., demographic, clinical, social) and information on exposures and potential confounders.
		(b) Indicate number of participants with missing data for each variable of interest.
		(c) Summarise follow-up time (e.g., average and total amount). Location (Pages 2-5, Results): (a) Detailed demographic and clinical characteristics are provided. (b) The number of participants with missing data is explicitly stated for ethnicity (n=12) and prior regimens (n=7). (c) Follow-up time is summarised: “Median follow-up was 9 months (IQR 5-19 months).”
Outcome data	15*	Report numbers of outcome events or summary measures over time. Location (Pages 3 and 5, Results, Paragraph 1 and Figure 3): The primary outcome (reasons for switch) is reported in detail with counts and percentages for each category (e.g., simplification n=169, 43.8%; toxicity n=115, 29.8%).
Main results	16	(a) Give unadjusted estimates and, if applicable, confounder-adjusted estimates and their precision (e.g., 95% confidence interval). Make clear which confounders were adjusted for and why they were included.
		(b) Report category boundaries when continuous variables were categorized.
		(c) If relevant, consider translating estimates of relative risk into absolute risk for a meaningful time period. Location (Pages 2-5, Results): (a) All results are presented as unadjusted, descriptive statistics (counts, percentages, medians), consistent with the study’s aim. (b) No continuous variables were categorised. (c) Not applicable.
Other analyses	17	Report other analyses done, e.g., analyses of subgroups and interactions, and sensitivity analyses. Location (Page 5, Results, Final Paragraph): A key secondary analysis is reported, comparing the number of switches before (n=74) and after (n=168) the implementation of the national procurement policy.
Discussion		
Key results	18	Summarise key results with reference to study objectives. Location (Page 5, Discussion, Paragraph 1): The discussion begins by summarising the main findings in direct relation to the study’s objectives. “The main results showed that the primary reason for switching…was simplification/rationalisation (43.8%), followed by toxicity (29.8%).”
Limitations	19	Discuss limitations of the study, taking into account sources of potential bias or imprecision. Discuss both direction and magnitude of any potential bias. Location (Page 6, Discussion, Paragraph 8 and Final Paragraph): The limitations section explicitly discusses the retrospective design, single-centre nature, and short follow-up. The subsequent paragraph explicitly names potential selection bias, information bias, and confounding.
Interpretation	20	Give a cautious overall interpretation of results considering objectives, limitations, multiplicity of analyses, results from similar studies, and other relevant evidence. Location (Page 5-7, Discussion): The entire section provides a balanced interpretation of the findings, contextualising them with other studies, clinical guidelines, and national policy, while acknowledging the study’s limitations.
Generalisability	21	Discuss the generalisability (external validity) of the study results. Location (Page 6, Discussion, Paragraph 8): Generalisability is explicitly addressed: “Being a single-centre study also means that the generalisability of our results may be limited.”
Other information		
Funding	22	Give the source of funding and the role of the funders for the present study and, if applicable, for the original study on which the present article is based. Location (Page 7, Additional Information, Disclosures): “All authors have declared that no financial support was received from any organisation for the submitted work,”
